# Rare Cause for Sudden Right Heart Failure

**DOI:** 10.1100/tsw.2010.190

**Published:** 2010-10-12

**Authors:** Amir M. Nia, Natig Gassanov, Matthias Schmidt, Ferdinand Kuhn-Régnier, Erland Erdmann, Fikret Er

**Affiliations:** ^1^ Department of Internal Medicine III, University of Cologne, Cologne, Germany; ^2^ Clinic of Nuclear Medicine, University of Cologne, Cologne, Germany; ^3^ Department of Cardiothoracic Surgery, University of Cologne, Cologne, Germany

**Keywords:** right heart failure, primary cardiac carcinoma, rhabdomyosarcoma, echocardiography

## Abstract

Right heart failure occurs daily in clinical settings, but an underlying cardiac malignant tumor is very uncommon. We report a case of a 48-year-old man presenting only with palpitations and decompensated heart failure. Echocardiographic imaging revealed a large tumor of the right ventricle. Shortly after a putatively successful surgical approach, the patient was admitted again with heart failure symptoms. On reassessment, a complete relapse with multiple metastases could be seen. Generally, cardiac malignant tumors are diagnosed at a time-point when therapeutic options are very limited or even postmortem. Broad echocardiographic screening in patients with unspecific symptoms might be helpful to detect cardiac malignant tumors at early stages.

## CASE

A 48-year-old, previously healthy man was admitted with acute decompensated right heart failure (RHF) causing massive lower extremity edema and dyspnea. The medical history was unremarkable except for paroxysmal cardiac palpitations for several months.

The cause for the acute right heart decompensation was identified in echocardiographic studies. Transthoracic and transesophageal echocardiography (TTE and TEE) revealed an obstructive structure embedded only within the right ventricle (RV), indicating a cardiac tumor (Fig. 1, Panel A; arrow). The complete staging did not identify other organ manifestations. Consequently, the tumor was removed surgically to eliminate the obstruction and to take specimens (Panel B). The subsequent pathologic assessment displayed a mainly undifferentiated spindle cell rhabdomyosarcoma. Consecutively, adjuvant chemotherapy was considered. The patient's condition improved rapidly and he was discharged 6 days after surgery.

Three weeks after cardiothoracic tumor resection, the patient was admitted again with recurrent RHF symptoms, prior to initiation of chemotherapy. Reassessment unfortunately revealed a large tumor, similar to the previously known sarcoma, in the lateral RV (Panel C). Contrast-medium computed tomography (CT) as well as total body positron emission tomography (PET) with 18F-fluoro-2-deoxy-D-glucose (FDG) identified a complete relapse (Panels D and E)[1,2]. Due to physiological cerebral metabolic overlapping, PET only showed pulmonary FDG-avid lesions, while CT detected additional

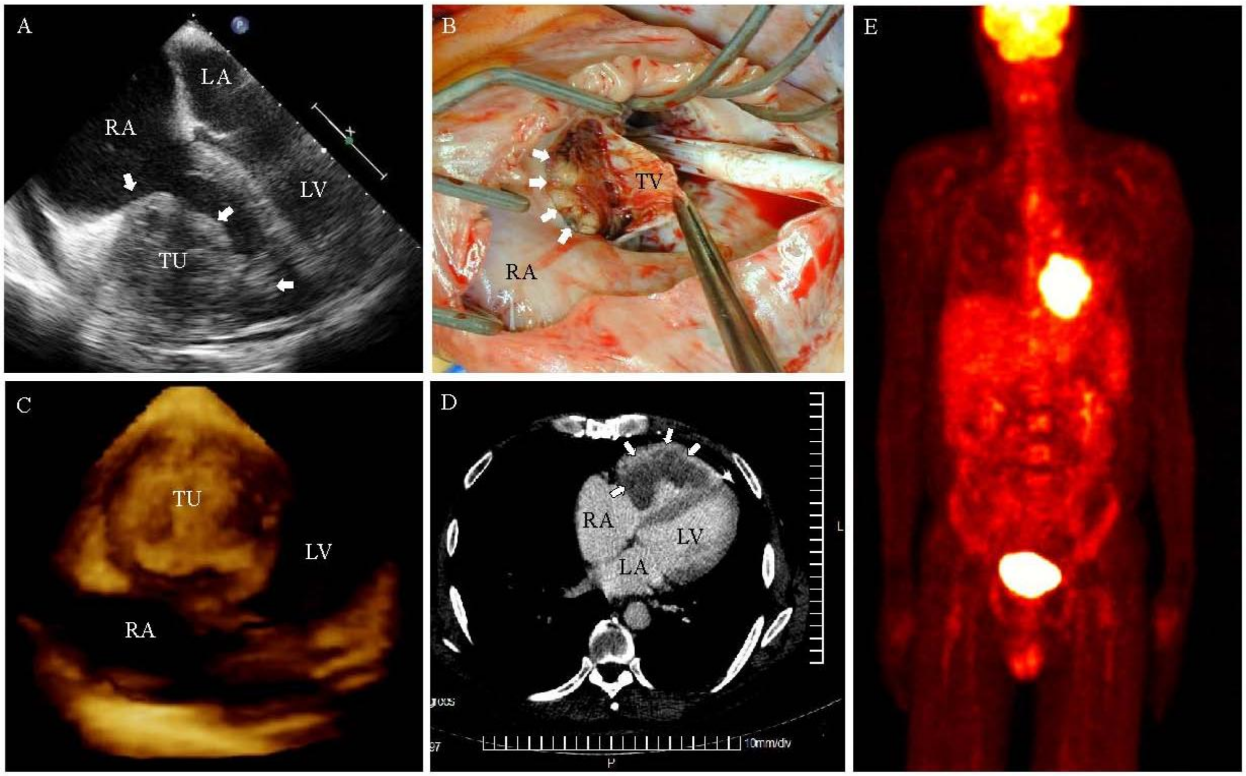

**FIGURE 1.** (A) TEE revealed a large tumor (arrowed) of the RV cavity (7 × 5 cm in size) without impairing the tricuspid valve. (B) Intraoperative imaging of the prolapsed mass (arrowed) confirmed tumor extension to the basis of the tricuspid valve. (C) 3-D TTE visualized an almost completely filled RV by the cardiac tumor. (D) Contrast-enhanced CT determined a renewed contrast agent–sparing tumor (arrowed) located in the RV. (E) Total body PET substantiated high-glucose metabolism of the cardiac carcinoma relapse by intensive FDG-uptake into the tumor (hot metal staining). TU indicates tumor, RA indicates right atrium, LA indicates left atrium, LV indicates left ventricle. TV indicates tricuspidal valve.

multiple brain metastases. Considering this devastating cancerous progression and the patient's wish, we discharged him to palliative care.

This striking case report impressively highlights that diagnosis of primary cardiac neoplasms is often made at the advanced stage because of the nonspecific nature of the symptoms that may suddenly exacerbate. Presentation of sudden illness with cardiac and constitutional symptoms or signs of systemic embolization may be susceptible for cardiac neoplasms[3,4]. Primary cardiac tumors are rare entities, but high percentages (~25%) of these are malignant tumors, mostly undifferentiated carcinomas, angiosarcomas, leiomyosarcomas, and rhabdomyosarcomas[5,6,7]. Larger prospective studies concerning management and treatment are missing[6]. The outcome in patients with malignant cardiac tumors is poor due to the limited therapeutic options and the late clinical manifestation, as demonstrated in the present case. The median survival is less than 1 year after initial diagnosis of a primary malignant cardiac tumor[5,6].

In contrast to primary cardiac tumors, metastatic involvement of the heart is over 20 times more common and has been reported in autopsy series in up to one in five patients dying of cancer[8,9]. In patients with isolated cardiac localization, allogenic heart transplantation may be meaningful as a potential curative approach[6,10]. For early diagnosis, a broad echocardiographic screening in patients with even mild cardiac symptoms, like palpitations as in this case, might be reasonable.
